# Metabolic therapy with PEG-arginase induces a sustained complete remission in immunotherapy-resistant melanoma

**DOI:** 10.1186/s13045-018-0612-6

**Published:** 2018-05-18

**Authors:** Carmela De Santo, Paul Cheng, Andrew Beggs, Sharon Egan, Alberto Bessudo, Francis Mussai

**Affiliations:** 1 Institute of Immunology and Immunotherapy, University of Birmingham, Birmingham, UK; 2 Bio-cancer Treatment International, Ltd., Shatin, Hong Kong; 3 Institute of Cancer and Genomic Sciences, University of Birmingham, Birmingham, UK; 4 School of Veterinary Medicine and Science, University of Nottingham, Sutton Bonington Campus, Sutton Bonington, UK; 5 Scripps Memorial Hospital, Encinitas, CA USA

**Keywords:** Arginase, Melanoma, Immunotherapy, Metabolism, BCT-100

## Abstract

**Background:**

Metastatic melanoma is an aggressive skin cancer with a poor prognosis. Current treatment strategies for high-stage melanoma are based around the use of immunotherapy with immune checkpoint inhibitors such as anti-PDL1 or anti-CTLA4 antibodies to stimulate anti-cancer T cell responses, yet a number of patients will relapse and die of disease. Here, we report the first sustained complete remission in a patient with metastatic melanoma who failed two immunotherapy strategies, by targeting tumour arginine metabolism.

**Case presentation:**

A 65-year-old patient with metastatic melanoma who progressed through two immunotherapy strategies with immune checkpoint inhibitor antibodies was enrolled in a phase I study (NCT02285101) and treated with 2 mg/kg intravenously, weekly pegylated recombinant arginase (BCT-100). The patient experienced no toxicities > grade 2 and entered a complete remission which is sustained for over 30 months. RNA-sequencing identified a number of transcriptomic pathway alterations compared to control samples. The tumour had absent expression of the recycling enzymes argininosuccinate synthetase (ASS) and ornithine transcarbamylase (OTC) indicating a state of arginine auxotrophy, which was reconfirmed by immunohistochemistry, and validation in a larger cohort of melanoma tumour samples.

**Conclusions:**

Targeting arginine metabolism with therapeutic arginase in arginine auxotrophic melanoma can be an effective salvage for the treatment of patients who fail immunotherapy.

## Background

Melanoma is an aggressive skin malignancy with increasing incidence, particularly among young adults. The identification of metastatic disease in lymph nodes or viscera at diagnosis is a poor prognostic factor [1]. Melanoma is well recognised as having a unique interaction with the immune response, both in terms of mechanisms of immune escape but also as a source of novel target antigens for the development of immune therapies [2]. Current treatment strategies for high-stage melanoma are based around the use of immunotherapy with immune checkpoint inhibitors such as anti-PDL1 or anti-CTLA4 antibodies to stimulate anti-cancer T cell responses, or signal-transduction inhibitors such as those targeting BRAF and MEK pathways [3, 4]. Despite these approaches, significant numbers of patients will still relapse and die of disease within 5 years [5]. Therefore, new strategies which target a different aspect of melanoma biology are needed.

Arginine is a semi-essential amino acid that is catabolised by tumour cell arginase I (ARGI) and II (ARGII) enzymes and nitric oxide synthetase (NOS2) into metabolites essential for cell proliferation, protein production and cell signalling [6]. However, defects in the expression of the arginine recycling enzymes ornithine transcarbamylase (OTC) and argininosuccinate synthetase (ASS) can result in a dependence of cancer cells on extracellular sources of arginine for survival [7].

The PEGylated recombinant human arginase, BCT-100, catabolises arginine into ornithine and urea [8]. Therapeutic administration leads to a systemic depletion of arginine in the blood and tissues providing a new approach to target cancer metabolism [9]. BCT-100 has shown safety and activity in patients treated with advanced hepatocellular carcinoma in early phase clinical trials [10]. Grade 3 serious adverse events (SAE) attributable to BCT-100 in these liver patients were limited to transaminitis or tumour pain in up to 15% of patients. Preclinical studies support the rationale for recombinant arginase therapy in melanoma; however, targeting arginine metabolism has not yet been considered an approach after failure of immunotherapy [11]. Here, we report a durable complete response by BCT-100 Arginase, in a patient with multiple recurrent melanoma lesions.

## Case presentation

A 65-year-old Caucasian male, a cigarette smoker of 50 pack-years, presented with dysphasia. A computed tomography (CT) revealed two 2 cm lesions in the left parietal lobe and bilateral enlarged axillary lymph nodes, with multiple pleural infiltrates in both lung apices, diaphragms and mediastinum. The patient previously had multiple pigmented skin lesions excised which were reported to be malignant. The brain lesions were completely excised and found to be vimentin and S100 positive on immunohistochemistry, confirming the diagnosis of malignant melanoma. The patient had no actionable BRAF V600E mutations (wild-type).

**Fig. 1:**
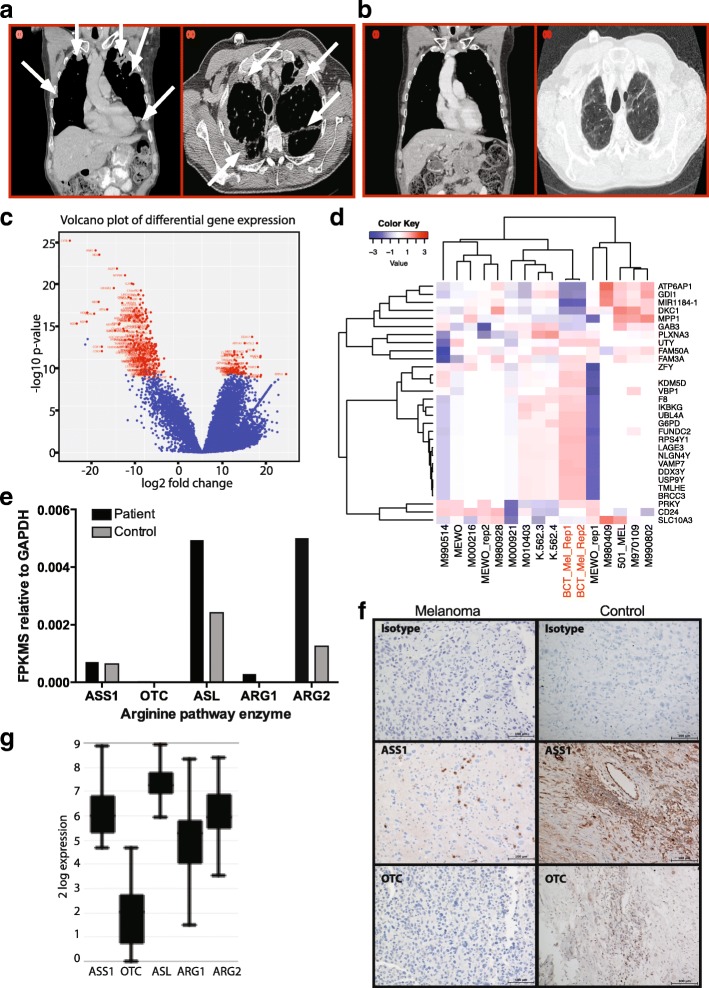
Arginase therapy is effective against immunotherapy-resistant melanoma. **a** CT thorax and abdominal radiographs at screening, showing extensive apical, pleural and diaphragmatic disease, in axial and coronal reconstructions and **b** complete remission in axial and coronal reconstructions. Radiographs were reported according to RECIST criteria. **c** Volcano plot of differential expression between complete responder melanoma and melanoma cohort. Red dots indicate significantly differential expression (BF > 10). Gene names shown. **d** Heatmap of top 30 differentially expressed genes via RNA-seq between BCT-100 responsive melanoma (BCT-Mel, *n* = 2 repeats, red) and control cohort (consisting of melanoma cell lines MEWO, 501_MEL and eight primary cell cultures from stage IV melanomas) as listed on the *X*-axis. **e** Expression of arginine pathway genes in patient and control samples, expressed relative to GAPDH. **f** Representative immunohistochemistry of patient’s tumour showing background staining (top), ASS (1:5000, dilution - middle) and OTC (1:5000 dilution bottom). Positive control staining on a neuroblastoma tumour shown for comparison. **g** Expression of arginine pathway enzymes in 44 samples of metastatic melanomas held with the R2: Genomics Analysis and Visualization Platform (http://r2.amc.nl)

The patient was initially treated with anti-CTLA4 antibody (Ipilimumab) for 3 months achieving a best response of stable disease, before progression of his chest lesions. He was then treated with anti-PDL1 antibody (pembrolizumab) for 2 months. The best response attained was a partial response; however, he went on to develop increasing pleural and axillary disease (Fig. 1a). Following a 3-month wash-out period, the patient consented to the clinical trial of BCT-100 therapy (NCT02285101).

Arginase (BCT-100) therapy was initiated at the established Recommended Phase II Dose (2 mg/kg intravenously weekly) that induces a persistent depletion in systemic arginine to < 8 μM. CT evaluation (week 13) revealed a partial response in all pleural-based lesions and therapy was continued. Restaging 20 weeks after the first dose of BCT-100 dramatically revealed a complete remission with clearance of all pleural-based and axillary disease. Thirty months from commencement of BCT-100, the patient remains on weekly dosing and in radiologically confirmed complete remission at all sites of disease (Fig. 1b). BCT-100 has been well tolerated with the patient experiencing only nausea, skin rash and diarrhoea, which were not deemed drug-related. He had no dysphasia since excision of the brain lesions. He continues to tolerate treatment with no SAEs greater than grade II.

Tumour tissue from the relapse surgery was evaluated by RNA-sequencing and compared to a control dataset obtained from the Genetic Expression Omnibus (GSE20156). Volcano plot shows significant differential expression between the melanoma patient treated and the control cohort (Fig. 1c). The 30 top differentially expressed genes are shown in Fig. 1d. Specific analysis of the arginine metabolic pathway identified that the tumour had relatively higher expression of arginase II catabolic enzyme to control melanomas. Expression of the recycling enzymes ASS and OTC expression were extremely low in all samples (Fig. 1e). Immunohistochemistry confirmed the almost complete absence of ASS and OTC enzymes, consistent with a state of arginine auxotrophy that defines sensitivity to arginase therapy (Fig. 1f). Interrogation of a further 44 samples of metastatic melanomas held with the R2: Genomics Analysis and Visualization Platform (http://r2.amc.nl) confirms the majority of tumours have lower OTC than ASS expression (Fig. 1g).

## Discussion and conclusions

This is the first report of a complete response in a metastatic melanoma patient treated with arginine deprivation therapy, notably in the setting of prior immunotherapy failure. The activity of PEGylated recombinant arginase has been reported to inhibit melanoma proliferation in S and G2/M phase and induce apoptosis in in vitro. Growth of melanoma cell line xenografts was significantly inhibited [11]. Among other tumour types, BCT-100 has demonstrated preclinical activity, and clinical responses have been seen in two clinical trials in adults with advanced hepatocellular carcinoma (HCC) [9, 10, 12]. The patient experienced no drug limiting toxicity or immunogenicity even after more than 2 years of weekly treatment. Identification of patients who are likely to respond to arginase therapy is a key challenge. The arginine recycling cascade is composed at the cellular level of the enzymes ASS, OTC and ASL, and expression determines cellular arginine auxotrophism [6]. Despite ASS or OTC having variable expression in a range of solid and haematological tumours, no ASL negative tumour cells have been described. Classically arginine depleting therapies such as pegylated arginine deiminase or BCT-100 are most effective when one or more of the enzymes have low or absent expression [8, 13]. Interestingly, BCT-100 also has activity against tumours which express both ASS and OTC, unlike ADI-PEG, suggesting differences in their modes of action or other intracellular pathways are contributing to determine cell fate [7, 9, 10, 12].

The transcriptional profile (as demonstrated by the heatmap in Fig. 1d) is completely different to that of a representative population of melanomas, suggesting that there may be distinct changes in this patients’ melanoma. Analysis of the patient’s tumour and control melanomas highlighted low ASS and OTC expression consistent with the paradigm that the absence of these enzymes renders tumour cells susceptible to arginine deprivation. Thus, melanomas may be broadly sensitive to arginine deprivation, and selection of patients based on ASS and OTC expression could be considered at screening in future clinical trials of arginase therapy with BCT-100. Interestingly, BCT-100 has demonstrated preclinical activity against melanoma cancer cells. Future translational studies on melanoma patients will investigate the threshold of ASS and OTC expression, and non-canonical pathways that could lead to resistance against arginase therapy.

Immunotherapy, notably immune checkpoint inhibitors, has heralded a sea-change in the management of adult malignancies, particularly in melanoma. However, many patients are still not cured even after addition of standard chemotherapy drugs or small molecule inhibitors to treatment regimens. Arginase therapy could see the advent of metabolic treatments for melanoma, following the longstanding success of targeting tumour metabolism in paediatric malignancies with drugs such as PEG-asparaginase and methotrexate. Importantly, the promising lack of toxicity of arginase therapy could allow this type of approach to be used in patients with significant co-morbidities which are frequently present in adult and elderly populations. In particular, BCT-100’s low toxicity profile has allowed for long-term administration with continued efficacy, compared to ADI-PEG or AEB1102 for which concerns over immunogenicity that result in allergic reactions or neutralising antibody development, or lack of activity have limited their use [9, 10, 13]. Indeed in a phase I/II trial of PEG-arginine deiminase in 31 patients with advanced, previously treated melanomas, no objective responses were seen and arginine depletion occurred only transiently [14]. In a phase I trial of AEB1102 which included nine patients with relapsed melanoma post immunotherapy with immune checkpoint inhibitors, the best response achieved was in one patient with stable disease (7 progressive disease, 1 unknown) [15]. The case also highlights the potential that initial priming of the immune response with anti-CTLA-4/ PD-1 checkpoint inhibitor antibodies may work in concert with subsequent BCT-100 in the setting of melanoma.

In conclusion, BCT-100 PEGylated recombinant human arginase led to a sustained and well-tolerated complete remission of a metastatic melanoma that failed prior immunotherapy, with the identification that tumour ASS and OTC expression could help future patient selection.
